# High Arctic ecosystem states: Conceptual models of vegetation change to guide long-term monitoring and research

**DOI:** 10.1007/s13280-019-01310-x

**Published:** 2020-01-18

**Authors:** Virve Ravolainen, Eeva M. Soininen, Ingibjörg Svala Jónsdóttir, Isabell Eischeid, Mads Forchhammer, René van der Wal, Åshild Ø. Pedersen

**Affiliations:** 1 Norwegian Polar Institute, Fram Centre, 9296 Tromsø, Norway; 2 UiT, The Arctic University of Norway, 9037 Tromsø, Norway; 3 University of Iceland, 101 Reykjavik, Iceland; 4 The University Centre in Svalbard, 9171 Longyearbyen, Norway; 5 The Centre for Macroecology, Evolution and Climate (CMEC) and Greenland Perspective (GP), Natural History Museum of Denmark, University of Copenhagen, Copenhagen, Denmark; 6 Department of Ecology, Swedish University of Agricultural Sciences (SLU), Ulls väg 16, 75651 Uppsala, Sweden; 7 University of Aberdeen, AB24 3UU Aberdeen, Scotland; 8 Norwegian Polar Institute, Fram Centre, 9062 Tromsø, Norway

**Keywords:** Arctic tundra, Climate change, Ecological monitoring, Ecosystem state, Press driver, Pulse driver

## Abstract

Vegetation change has consequences for terrestrial ecosystem structure and functioning and may involve climate feedbacks. Hence, when monitoring ecosystem states and changes thereof, the vegetation is often a primary monitoring target. Here, we summarize current understanding of vegetation change in the High Arctic—the World’s most rapidly warming region—in the context of ecosystem monitoring. To foster development of deployable monitoring strategies, we categorize different kinds of drivers (disturbances or stresses) of vegetation change either as pulse (i.e. drivers that occur as sudden and short events, though their effects may be long lasting) or press (i.e. drivers where change in conditions remains in place for a prolonged period, or slowly increases in pressure). To account for the great heterogeneity in vegetation responses to climate change and other drivers, we stress the need for increased use of ecosystem-specific conceptual models to guide monitoring and ecological studies in the Arctic. We discuss a conceptual model with three hypothesized alternative vegetation states characterized by mosses, herbaceous plants, and bare ground patches, respectively. We use moss-graminoid tundra of Svalbard as a case study to discuss the documented and potential impacts of different drivers on the possible transitions between those states. Our current understanding points to likely additive effects of herbivores and a warming climate, driving this ecosystem from a moss-dominated state with cool soils, shallow active layer and slow nutrient cycling to an ecosystem with warmer soil, deeper permafrost thaw, and faster nutrient cycling. Herbaceous-dominated vegetation and (patchy) bare ground would present two states in response to those drivers. Conceptual models are an operational tool to focus monitoring efforts towards management needs and identify the most pressing scientific questions. We promote greater use of conceptual models in conjunction with a state-and-transition framework in monitoring to ensure fit for purpose approaches. Defined expectations of the focal systems’ responses to different drivers also facilitate linking local and regional monitoring efforts to international initiatives, such as the Circumpolar Biodiversity Monitoring Program.

## Introduction

Vegetation plays a key role in terrestrial ecosystem functioning, with its attributes such as species composition, structure, and productivity influencing soil carbon and nitrogen cycling and supporting associated biodiversity (Wookey et al. 2009). International assessments such as the Circumpolar Biodiversity Monitoring Plan (CBMP within CAFF—Conservation of Arctic Flora and Fauna) highlight the importance of monitoring vegetation (Christensen et al. 2013; Ims and Ehrich 2013). The CBMP has suggested four Focal Ecosystem Components for monitoring plants: (i) all plants (species, life-form groups and associated communities); (ii) rare species and species of concern; (iii) non-native species; and (iv) species that humans use as food. Abundance, productivity, composition, diversity, and phenology are attributes that further specify the monitoring of most of these Focal Ecosystem Components. These attributes describe vegetation characteristics that are commonly used to measure shifts in whole ecosystem structure and function, i.e. ecosystem state shifts (Scheffer and Carpenter 2003; Bråthen et al. 2017).

Adaptive monitoring is chosen as the paradigm to be used in CBMP initiatives (Christensen et al. 2013). In this framework, conceptual models form a basis for hypotheses and predictions about change. The process of describing the expected changes often allows for the identification of variables to monitor (Lindenmayer and Likens 2009). Conceptual models are therefore a useful tool to inform monitoring decisions. Establishment of adaptive monitoring programmes typically concerns (i) articulation of the monitoring targets set as questions or hypotheses about the system’s change; (ii) designing a monitoring approach and deciding upon the variables; and (iii) performing data collection and analysis. Thereafter, interpretation of the results will reveal the need to re-visit the questions and adjustment of protocols. Adaptivity refers to the programmes possibility to adjust to new potentially important processes, while maintaining the integrity of core variable sets. Hence, adaptive monitoring is not at odds with maintaining long time-series, but rather articulates a way to adapt new protocols and measures as needed (Lindenmayer et al. 2011).

One way to conceptualize vegetation change is the ‘state-and-transition’ or ‘alternative stable states’ approach (Briske et al. 2008). The premise of the alternative states models—a term we prefer given that many states are transient rather than stable (Fukami and Nakajima 2011) is that a given location or habitat may occur in one or more different vegetation states depending on conditions (i.e. ‘driver impacts’). Hypothesized alternative states models can hence be used as a tool in building conceptual models that guide monitoring. State transition models developed for rangelands are an example of this, with vegetation structural components and the drivers behind changes specified to produce comprehensive catalogues of alternative states and their transitions (Stringham et al. 2003; Briske et al. 2005; Barrio et al. 2018). In tundra ecosystems, some alternative state models have been proposed (Van der Wal 2006; Bråthen et al. 2017; Barrio et al. 2018), but the use of specific conceptual models in Arctic monitoring programmes has been very limited. However, the question “how does vegetation change” is put in the spotlight by on-going rapid climate change (Anisimov et al. 2007; Post et al. 2009; Christensen et al. 2013), and calls for increased attention to what suite of interacting biotic and abiotic drivers are key when developing vegetation monitoring strategies.

Vegetation change may happen gradually or abruptly. Drivers of vegetation change can likewise manifest themselves as a trend developing gradually over time, or as a sudden event that pushes the subject of interest over a threshold into another domain (Briske et al. 2008). Indeed, Arctic climate change provides examples of drivers that induce both gradual change (e.g. rising mean temperature) and discrete events that occur suddenly (e.g. mild winters with rain-on-snow events or other weather extremes) (Anisimov et al. 2007). In other words, Arctic climate change generates disturbances or stresses that can manifest themselves as ‘press driver’ (i.e. disturbances or stresses that remain in place for a long time, or slowly increase in pressure) and those that act as ‘pulse driver’ (i.e. sudden and short events, though their effects may be long lasting). A press driver can be described as extensive, pervasive, or subtle and a pulse driver as infrequent, sudden or as an event (Collins et al. 2011; Ratajczak et al. 2017). Current understanding of what shapes Arctic vegetation acknowledges the influence of what can be termed press and pulse drivers (Walker et al. 2005; Zimov 2005; Van der Wal 2006; Wookey et al. 2009; Myers-Smith et al. 2011; Bråthen et al. 2017), but the last decades of rapid changes in climate warrant discussion of new conceptual models that express their distinction more clearly.

Arctic land areas are warming considerably and are at risk of experiencing ecosystem change and biome shifts at already relatively modest increases in global mean temperatures (Beck et al. 2011; Grimm et al. 2013; Warszawski et al. 2013). However, evidence is accumulating that vegetation change in the Arctic is highly spatially heterogeneous (Beck and Goetz 2011; Elmendorf et al. 2012a; Myers-Smith et al. 2015; Huang et al. 2017) and lagging behind temperature change (Huang et al. 2017). Indeed, a review of experimental warming studies and long-term monitoring in the Arctic showed that no change in plant abundance was the most common response (Bjorkman et al. 2020). High Arctic, sensu Christensen et al. (2020), vegetation abundance is characterized by no or weak trends in relation to experimental warming and ambient rising temperatures (Hudson and Henry 2010; Prach et al. 2010; Elmendorf et al. 2012a). This heterogeneity in vegetation responses to warming challenges monitoring to strike a balance between ecosystem-specific understanding and general understanding of tundra vegetation changes that can be applied more universally.

Here, we propose a general and a detailed conceptual model for vegetation change in Svalbard that can help guide and inspire vegetation monitoring and future research also in other High Arctic tundra ecosystems. Some of the impact pathways discussed below have been documented in previous research, while others are proposed as hypotheses to be tested in future research and through monitoring. For now, we take a pragmatic approach, and categorize drivers as ‘press’ when their pressure gradually increases, or remains in place, over multiple years and as ‘pulse’ when they change over less than annual timescales, acknowledging that the best definition may vary between subjects of interest. We discuss press and pulse drivers in moss-graminoid tundra habitats in three potential alternative vegetation states characterized by (i) a thick moss layer, (ii) herbaceous plants, and (iii) bare patches. Because there is more monitoring knowledge from the Low Arctic than the High Arctic (Bjorkman et al. 2020), we build on insight from lower latitude but focus on the High Arctic. We hope our examples will spark a broader and more thorough discussion on the full range of High Arctic vegetation states and drivers of transitions between them.

## Drivers of Arctic vegetation change: current examples of press and pulse drivers

Average temperature rise is an example of a mainly press driver in the system. A number of studies have found that in the Low Arctic warmer temperatures over time influence plant growth and abundance, especially, shrubs have increased (Tape et al. 2006; Elmendorf et al. 2012b; Myers-Smith et al. 2015; Bråthen et al. 2017). Tall shrubs are a growth form that is lacking in the High Arctic where dwarf shrubs and herbaceous plants are the main constituents of vegetation. Yet, the biomass of High Arctic plants is also closely linked to summer temperatures (Schmidt et al. 2012; Van der Wal and Stien 2014; Myers-Smith et al. 2019). With a continued press from warmer summer temperatures, plant abundance of especially the relatively fast growing growth forms such as the herbaceous forbs and graminoids, as well as the woody, deciduous shrubs would be expected to increase (Elmendorf et al. 2012b).

Sudden or short-term pulse drivers can damage vegetation, creating bare ground in previously vegetated habitats. Contrary to boreal and temperate ecosystems where fire, drought and insect outbreaks cause sudden, large-scale state shifts (Scheffer and Carpenter 2003; Briske et al. 2005; Beck et al. 2011), Arctic tundra has not been characterized by such dramatic and spatially extensive responses to these pulse drivers. However, climate warming may potentially change this. Tundra fires (Mack et al. 2011) and variable winter weather causing basal ground-ice formation (Bokhorst et al. 2012; Milner et al. 2016; Peeters et al. 2019) are already documented examples of pulse events that are likely to play a more prominent role for tundra vegetation in the future. Small rodent population fluctuations and their impact when at high densities act as a pulse driver of vegetation composition in many Low Arctic ecosystems (Ravolainen et al. 2011; Olofsson et al. 2012), and in some High Arctic systems (Johnson et al. 2011; Bilodeau et al. 2014). Yet, documentation of their potential to create persistent bare ground patches is lacking.

The same factor, such as grazing, may act as a press or pulse driver depending on its temporal pattern of change. A sudden decrease in grazing pressure could, for instance, shift herbaceous grassland to woody tundra. This has been documented in riparian Low Arctic tundra where cessation of grazing led to surprisingly fast increases in willow growth (Ravolainen et al. 2014), suggesting that here reindeer (*Rangifer tarandus*) grazing acted as a pulse driver. On the other hand, sustained grazing pressure—a press driver—in the same study system keeps willow shrub recruits restricted to a low height and restricts the altitudinal limit of tall willow shrubs (Bråthen et al. 2017).

## Svalbard vegetation states in moss-graminoid tundra

**Fig. 1:**
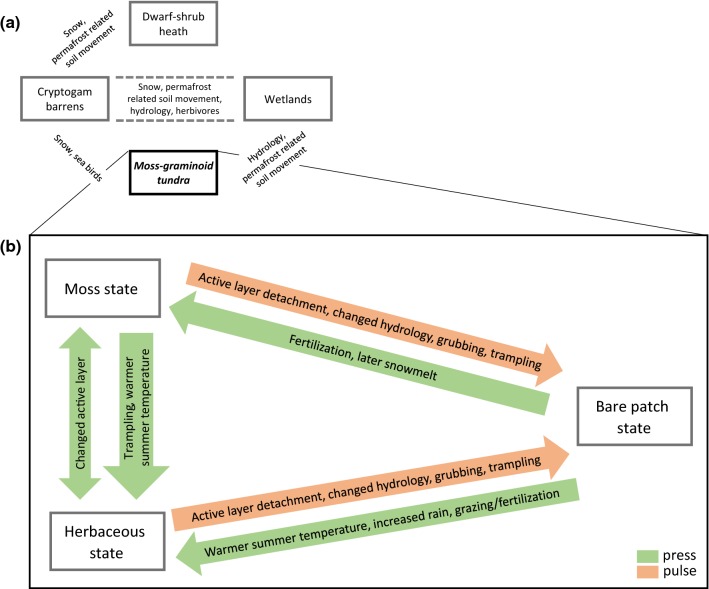
Topography, snow cover, hydrology, herbivory, and substrate are general factors supplemental to climate that differentiate High Arctic habitat types on Svalbard: wetlands, dwarf-shrub heathlands, barrens with lichens or mosses, and moss-graminoid tundra (**a**). Within the context given by the general habitats, transitions between alternative states can happen (**b**). We suggest the moss-graminoid tundra can be found in a (i) moss, (ii) herbaceous, (iii) or bare patch characterized state. The drivers that cause shifts between these states can be characterized as those that gradually change their impact (‘press’), and those whose impact is a sudden event (‘pulse’). Both biotic and abiotic drivers can push the moss-graminoid tundra in the same direction, e.g. both sudden active layer detachments and high abundance of herbivores trampling or grubbing can cause the shift from the vegetated to the bare patch state. See main text for examples and references

Differences in topography, snow lie, hydrology, and substrate give rise to general habitat types such as wetland, dwarf-shrub heath, cryptogam-barren and moss-graminoid tundra in Svalbard (Fig. 1a). While dramatic environmental changes can ultimately drive shifts from one of these broad types to another, here we will focus mainly on examples from Svalbard moss-graminoid tundra which covers 8–24% of continuous vegetation in central Spitsbergen (Johansen et al. 2012) and is a key habitat for many terrestrial animals (Staaland et al. 1993; Speed et al. 2009).

We propose a generalized conceptual model with three hypothesized alternative states within moss-graminoid tundra (Fig. 1b). Biotic and abiotic drivers may act in concert and push moss-graminoid tundra towards similar state changes. For the transition between the moss state and the herbaceous state, we hypothesize mainly press driver impacts. A warmer climate, gradually increased active layer depth, and more available nutrients could push the moss state towards the herbaceous state, with the latter sustaining a greater number of herbivores (Fig. 1b). The same state transition, with increased graminoid dominance, is also likely to occur where large herbivores graze, trample, and fertilize vegetation, i.e. in response to increased herbivore numbers (Van der Wal 2006). The opposite transition, from the herbaceous state to the moss state, could potentially occur with reduced herbivore activity. Graminoids and forbs are already common in many High Arctic vegetation types (Walker et al. 2005). We hypothesize that the herbaceous, graminoid-dominated state, with relatively fast nutrient cycling and high tolerance to mechanical disturbance and grazing, would become more common given a press from longer, warmer, and wetter summers in the presence of a high number of herbivores, notably reindeer.

Abiotic events and biotic agents can act as pulse drivers to shift the moss or the herbaceous state to the state characterized by bare patches (typically from < 1 m^2^ to covering a few 10 m^2^, and often occurring over large areas) (Fig. 1b). In the High Arctic, warm summers cause active layer deepening, thaw slumps and the opening up of bare soil (Anisimov et al. 2007; Lousada et al. 2018), and this can happen in the course of few weeks or during a single summer season (Ravolainen pers.obs.). In the Canadian Low Arctic, lesser and greater snow goose grubbing, i.e. foraging for below-ground plant parts, has caused local and large-scale shifts to a bare ground state in interaction with hydrology and salinity (Jefferies et al. 2006; Lefebvre et al. 2017). Goose foraging can remove nearly all vegetation within only a few years also in the High Arctic (pers. obs. authors). Hence, although goose populations may gradually increase, being a ‘press driver’, goose grubbing at a given location, due to an amplifying effect of saline sub-soil or other abiotic factors, may act as a pulse driver. We hypothesize that the abiotic and biotic pulse drivers could increase the number and size of bare patches, causing a distinct bare ground state in landscapes that currently have continuous plant cover and hence changing the spatial patterning of vegetation.

**Fig. 2:**
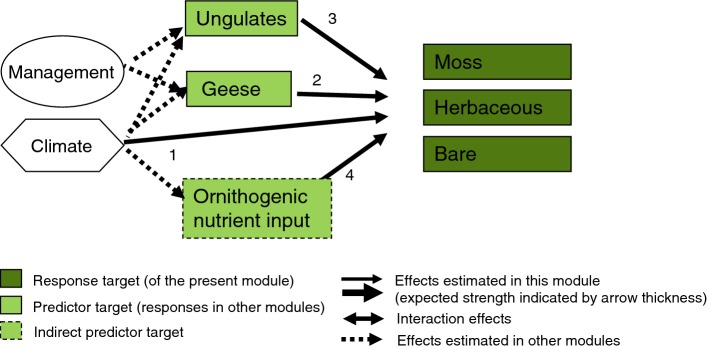
A detailed conceptual model for moss tundra on Svalbard implemented within the monitoring programme Climate-ecological Observatory for Arctic Tundra—COAT. The included drivers are expected to have direct impact on the state shifts. Indirect impacts (dashed lines) and effects the vegetation can have on the herbivores have been outlined earlier (Ims et al. 2013). Climate (pathway 1) can act as a ‘press’ via gradually warming temperature, or as a ‘pulse driver’ through, for example, abrupt extreme winter weather events. Likewise, the impact of herbivores can happen as an abrupt pulse event, as in the case of goose grubbing driving vegetation patches from vegetated to the bare patch state (pathway 2), or as press herbivory by reindeer gradually causing a shift from the moss to the herbaceous state (pathway 3). Fertilization by seabirds is an important driver of state shifts on the coast (pathway 4). See main text for more examples and references

While a general model such as presented in Fig. 1b is useful for clarifying which state transitions can be expected to occur, a monitoring programme is reliant on an integrated effort that specifies how to monitor both the vegetation state shifts and the drivers. In the following, we suggest a more detailed conceptual model that specifies expected climate- and management-driven impacts of herbivores and nutrients on vegetation state transitions in moss-graminoid tundra. In doing so, we illustrate the process from depicting conceptual models to implementing practical monitoring with the required set of variables (Fig. 2, Table 1). The model suggested here is a part of an ecosystem-based, adaptive monitoring programme in Norway (Ims et al. 2013).

**Table 1:** The set of variables derived from the conceptual model for monitoring of vegetation state transitions in moss-graminoid tundra on Svalbard (Fig. 2). Path refers to the pathways outlined in Fig. 2. The relation to the Circumpolar Biodiversity Monitoring Program (CBMP) for terrestrial Arctic and the Focal Ecosystem Components (FEC), and their Attributes (Attr.) are indicated

State variable	Interval	Method	Path	FEC*	Attr.*
Moss layer thickness	1 year	Field measure			
Biomass of vascular plant species and functional groups	1 year	Point frequency		All plants	Diversity, composition, and abundance
Ice damage	1 year	Transect, drone and satellite imagery	1		
Extent of vegetation types, bare ground	5 years	Drone and satellite imagery	2, 3	All plants	Diversity, composition and abundance/diversity and spatial structure
Productivity at peak season	1 year	Drone and satellite imagery, NDVI	1	All plants	Productivity
Phenology	1 year	Drone and satellite imagery, time-integrated NDVI	1	All plants	Phenology
Air and soil temperatures	Multiple	Weather stations, medium-sized station, small, distributed loggers	1		
Permafrost thaw depth	1 year	Late summer maximum depth at bore hole	1		
Snow depth, duration, distribution	Multiple	Field measurement, modelling	1		
Soil moisture	Multiple	Weather stations, small loggers	1		
Abundance of herbivores	1 year	Pellet counts, camera traps, population census	2, 3		
Grubbing impact	1 year	Counts	2, 3		
Soil nutrient level	1 year	Near-infra red spectrometry	2, 3, 4		
Ground-ice formation	Multiple	Field measurement, modelling	1		
Permafrost-soil movement	5 year	Satellite and drone imagery	1		

*The attributes of the focal ecosystem component in the Circumpolar Biodiversity Monitoring Plan; “All plants (species, life-form groups and associational communities) include attributes “diversity, composition and abundance”, “diversity and spatial structure”, “productivity”, and “phenology”

Svalbard is a High Arctic archipelago with a relatively simple food web. Moist, potentially very productive habitats of Svalbard tundra harbour moss tundra (Vanderpuye et al. 2002; Walker et al. 2005) that we suggested can exist in three alternative states: (i) dominated by mosses; (ii) herbaceous vascular plants in a matrix of moss, or (iii) as bare ground (Figs. 1, 2). The suggested moss-dominated state has a relatively high productivity and capability to retain nutrients and moisture. A deep moss layer, often with species from the genera *Aulacomnium*, *Tomentypnum*, and *Sanionia,* (Eurola and Hakala 1977; Vanderpuye et al. 2002; Van der Wal and Brooker 2004) insulates the soil, keeping it cool and with shallow active layer (Gornall et al. 2007). The suggested herbaceous state has abundant graminoids, e.g. *Alopecurus magellanica*, *Poa* spp., forbs, e.g. *Saxifraga* spp., *Bistorta vivipara,* and dwarf shrubs, particularly *Salix polaris*, growing in a moss matrix (Eurola and Hakala 1977; Vanderpuye et al. 2002). Soils tend to be warmer and the active layer is consequently deeper than in the moss-dominated state (Van der Wal and Brooker 2004). The critical functions of the vegetated states of moss tundra, supporting vertebrate communities and regulating ecosystem processes, depend on the level of grazing, manuring, and disturbance imposed by the herbivorous animals [Figs. 2, 3 (van der Wal et al. 2004; Van der Wal and Brooker 2004; Van der Wal 2006)], but also by fertilization from colony nesting birds (Eurola and Hakala 1977; Vanderpuye et al. 2002). The suggested bare ground state can be either exposed organic soil, including decomposing moss, or mineral soil (sandy, or silty); and bare areas can range from small (< 1 m^2^) to large (10’s of m^2^). In this state, the soil organic carbon content is lower than in the other two alternative states, and erosion and leaching may cause nutrient depletion (Van der Wal et al. 2007).

Summer temperature and soil moisture are likely the most important abiotic press drivers that affect moss tundra vegetation (Fig. 2, pathway 1). In Svalbard, summers have warmed moderately over the last 50 years, while spring, autumn and particularly winter temperatures have had a stronger warming trend (winter 1.6°C/decade) (Vikhamar-Schuler et al. 2016). We hypothesize that the warmer summers in central Spitsbergen during the last 50 years (Van Pelt et al. 2016) can, in the future, exert a press impact in favour of the herbaceous state over the moss-dominated state.

A climate-related pulse driver that influences the terrestrial ecosystem on Svalbard are rain-on-snow events in winter, leading to ground-ice formation (Fig. 2, pathway 1). The effects of ground-ice on plant communities have been mostly studied in dwarf shrub species that have sensitive organs exposed above-ground, revealing high spatial variability in damage from winter weather (Milner et al. 2016; Bjerke et al. 2017). The spatial extent and longer-term effects of winter damage to shrubs remains a question for future research and monitoring. The extent of winter damage in herbaceous and mossy vegetation states is currently undocumented. The frequency of “rain-on-snow” events has increased and the precipitation and snow cover patterns are expected to change (Adakudlu et al. 2019; Peeters et al. 2019). We therefore hypothesize that also plants with all sensitive organs below or at the soil surface may suffer from winter climate events, and integrate measurements of winter damage and extreme winter weather into the vegetation monitoring (Table 1).

It is hardly possible to predict climate impacts on moss tundra without considering the activities of herbivores, whose populations are dynamic and changing. Resident herbivores (Svalbard reindeer, *Rangifer tarandus platyrhynchus* and Svalbard rock ptarmigan, *Lagopus muta hyperborea*) are affected by changes in winter climate (Hansen et al. 2013; Albon et al. 2017). Mild winter weather (pulse driver), leading to ground-ice formation, results in reduced reindeer population growth rates (Albon et al. 2017). By contrast, longer and warmer summers (press driver), given they result in higher primary production, appear to increase population growth rates (Hansen et al. 2013). Similar positive effects of longer summers in the Arctic likely will act on the migratory geese (Jensen et al. 2008), whose populations have increased dramatically during the last decades because of reduced hunting pressure and greatly improved food availability in the wintering areas (Fox et al. 2005; Fox and Madsen 2017) .

A major way through which herbivores impact the vegetation is through physical disturbance (Fig. 2, pathways 2 and 3). Particularly, the moss-dominated state is sensitive to changes in disturbance by reindeer and geese (Van der Wal and Brooker 2004; Speed et al. 2009) (Fig. 2, pathway 2). In early spring and summer, pink-footed geese (*Anser brachyrhynchus*) grub for below-ground plant parts of particularly grasses (*Dupontia* spp.) and sedges (*Eriophorum scheuchzeri*), but also *Equisetum arvense* and *Bistorta vivipara*, disrupting the moss layer (Fox et al. 2006; Anderson et al. 2012). Pink-footed goose grubbing affects an increasing proportion of the vegetated ground, and the effect of their activities is found in nearly all vegetation and landscape types (Pedersen et al. 2013). Patches opened by the grubbing activity of geese are currently mostly small but highly frequent in the landscapes (nearly 50% of 20,000 m^2^ of moss tundra transects surveyed in 2018 had signs of grubbing disturbance; Ravolainen et al. unpublished data). Re-growth of moss is slow relative to vascular plants, which makes the moss state more sensitive to trampling than the herbaceous state. Timing of snowmelt modulates the impact of pink-footed geese on tundra vegetation since it controls the spatial distribution of feeding geese (Anderson et al. 2016). It is not known what degree of grubbing intensity leads to changes in hydrology and other local abiotic factors. Whether the extent of grubbing (see Speed et al. 2009 for spatial predictions) continues to increase, whether there is potential for it affecting food availability for the year-round resident herbivores, and how dynamic the different vegetation states are in their responses and recovery remain to be addressed in future work.

Thaw slumps, ice wedge polygon collapses, and active layer detachments are pulse driver disturbances related to permafrost processes occurring on Svalbard. Yet, their impacts on vegetation state shifts lack documentation. From other Arctic areas, we know that eroding gullies (Perreault et al. 2016) and permafrost thaw slumps (Sluijs et al. 2018) can influence landscape structure and vegetation as local pulse drivers. The bare ground created by permafrost-related processes differs from that opened by geese in one fundamental way: landslides, thaw slumps, and thermokarsts remove or move soils, while geese remove the vegetation while leaving the soil largely in place. Thus, the respective formation mechanism of the bare ground patches could lead to different plant recruitment potential. We therefore hypothesize that habitats disturbed by geese will in the future support different vegetation states than bare ground created by permafrost-related erosion.

Another press driver in moss tundra is increasing nutrient turnover (Fig. 2, pathways 2 and 3). Both geese and reindeer enhance nutrient turnover (Van der Wal and Hessen 2009; Sjogersten et al. 2010), but direct comparisons of their relative effects are lacking. What level of nutrient availability is necessary to maintain the different tundra states remains to be investigated. At high levels of nutrient input by seabirds moss tundra shifts to the herbaceous state (Eurola and Hakala 1977) (Fig. 2, pathway 4). Given that more than three million pairs of altogether 20 species of seabird breed on Svalbard, their influence should be considerable, as they are a significant driver of nutrient transfer from the marine to the terrestrial realm (Zwolicki et al. 2013). Vegetation development under climate change scenarios in moss tundra can be expected to be inherently tied to trends in seabird populations—particularly in coastal areas—in addition to the above-mentioned effects by herbivores.

Proportions of plant biomass consumed by reindeer across larger units of vegetation are generally low, and likely not dissimilar to estimates obtained for muskoxen *Ovibos moschatus* in NE Greenland although effects on vegetation are measurable when muskoxen are excluded (Mosbacher et al. 2016). In line with this, the few short-term exclosure studies of Svalbard reindeer herbivory have not found strong impacts on plant biomass (Wegener and Odasz-Albrigtsen 1998; Dormann et al. 2004). Studies on geese, on the other hand, have shown locally strong suppression of vascular plant biomass, which could ultimately drive the system to a moss-dominated state (for a review, see Van der Wal and Hessen 2009). Were intense grazing to cease, then the herbaceous state would re-emerge (Sjogersten et al. 2011). Studies looking at the cumulative effects of these key herbivores at current population levels are lacking. The reindeer population in Svalbard has doubled since 1980’s (Le Moullec et al. 2019) and the pink-footed goose population increased by 36% between 2007 and 2013 (Anderson et al. 2016). We can hypothesize that changing population sizes of reindeer and pink-footed geese, as they share spring and summer habitat and forage plants, may be an additive press driver, shifting the system from cryptogam to herbaceous state and potentially, if grazing pressure become high enough, to a bare ground state (Fig. 2 pathways 2 and 3).

## Arctic vegetation states: ecosystem-specific shifts

Warmer summer temperature (Schmidt et al. 2012), extreme winter weather (Bjerke et al. 2017), disturbance from herbivores (Olofsson et al. 2012), and permafrost-related processes, such as deeper thaw and active layer detachment (Myers-Smith et al. 2019), can all lead to vegetation state shifts. On a general level, we suggest that these drivers are important, and transitions towards more deciduous shrubs or more herbaceous vegetation at the cost of cryptogams can happen across the Arctic. However, we hypothesize that habitat differences (notably terrain, water regime, substrate, and snow distribution), as well as the assemblage of herbivores—of different-sized species with distinct feeding modes—will continue to dictate which states are possible and which transitions happen. For instance, the impact of very large herbivores like muskoxen will likely manifest partly through their trampling effect on mosses. Muskoxen trampling of wet habitats has been documented to decrease soil temperatures (Mosbacher et al. 2016), which is opposite to what has been observed in drier habitats concerning reindeer (Van der Wal and Brooker 2004; Van der Wal 2006). Further, bare ground patches caused by e.g. permafrost thaw slumps will in moist habitats probably revegetate within the course of some years (Lantz et al. 2009). Yet, we hypothesize that in drier habitats re-colonization might take longer and potentially even be hampered by increased frequency of extreme winter events such as ice encasement. These examples highlight the need for habitat and region specific, ecosystem-based conceptual models to guide future monitoring and research on Arctic vegetation states.

## Integration of drivers in vegetation monitoring

The implementation of monitoring following a conceptual model as outlined above requires establishment of integrated measures capturing vegetation ‘response variables’, as well as measurements of the respective drivers. The variables monitored in Svalbard moss tundra (Table 1) provide an example of how monitored variables link to the above-unfolded conceptual model (Fig. 2). The relative importance of grazing, trampling, grubbing, and abiotic disturbances as drivers of vegetation state shifts warranted the selection of variables to monitor. More work is needed before we fully understand what drivers are best categorized as pulse and press in the changing Arctic terrestrial ecosystems. Our focus here has been on climate and herbivores as drivers of vegetation state shifts, while impacts of vegetation change on the herbivores are described elsewhere in models focussed on reindeer and geese (Ims et al. 2013). While being specific to the focal ecosystem, the conceptual model and the variables presented here are at the same time in correspondence with the focal ecosystem components suggested for monitoring in the international Circumpolar Biodiversity Monitoring Plan within CAFF (Christensen et al. 2013). A difference between the concept models of the international plan and the models we outline here and in the Climate-ecological Observatory for Arctic Tundra—COAT (Ims et al. 2013) is that we explicitly describe expected directional impacts of individual drivers on tundra vegetation states and other ecosystem components such as the respective herbivores. This hopefully facilitates understanding of the linkages between the ecosystem components, in line with ambitions given in the CBMP plan (Christensen et al. 2013) and other international assessments (Ims and Ehrich 2013). Monitoring data that allow for directly linking the variables describing ecosystem state and drivers of state shifts can give insights into climate–ecosystem dynamics (Post et al. 2009; Ims et al. 2013).

Conceptual models should be re-visited at regular intervals to adapt them and the respective monitoring programme to new knowledge (Lindenmayer et al. 2011). For instance, changes in environmental conditions may suggest that new impact pathways have gained importance. Currently, the occurrence of alien plant species, identified as a Focal Ecosystem Component by CBMP (Christensen et al. 2013), is largely confined to Svalbard’s settlements (Alsos et al. 2015) similar to the situation in the circumpolar Arctic (Wasowicz et al. 2019). Should alien plant species become more frequent in natural habitats, then they and their drivers would be included in the monitoring. By contrast, variables that are central for monitoring climate change impacts on vegetation, such as weather variables and peak season biomass, are continuing time-series. While adjustments to the sampling protocols of these time-series will need to be considered at regular intervals, any change should be made without risking the integrity of long-term time-series. For instance, changes to new methods should be calibrated against previously used methods. One of the strengths of relying on the adaptive monitoring paradigm is the active consideration of new elements in context with the established programme, while maintaining time-series of core variables (Lindenmayer et al. 2011). Currently, very few monitoring programmes use adaptive monitoring, and we suggest that more active usage of this approach is warranted given the rapid change of Arctic ecosystems.

## Key findings and recommendations for the circumpolar biodiversity monitoring plan

Based on known and expected vegetation state changes in High Arctic Svalbard, we propose the use of conceptual models as basis for monitoring tundra vegetation. Current understanding of our worked up, and implemented case, moss-graminoid tundra, suggests that additive effects of a warmer climate and increasing herbivore pressure are likely to drive this system from a moss-dominated state with cool soils towards either a herbaceous state or a bare ground state, both having warmer soils and deeper active layers. We developed an ecosystem-specific conceptual model to determine the variables currently included in the long-term adaptive ecosystem monitoring on Svalbard. The examples of possible vegetation state transitions described for Svalbard moss-graminoid tundra are intended to encourage discussion and development of a broader set of conceptual models and potential vegetation states for other rapidly changing Arctic regions. We propose that development of a comprehensive set of conceptual models, with built-in best estimates of potential shifts in vegetation states, should become a priority of international monitoring bodies as they would help to link local and regional monitoring efforts in a circumpolar context.
